# The dermatology life quality index among healthcare workers during the COVID-19 pandemic

**DOI:** 10.1192/j.eurpsy.2023.1678

**Published:** 2023-07-19

**Authors:** I. Sellami, A. Abbes, H. Aicha, H. Halweni, A. Feki, M. L. Masmoudi, K. Jmal Hammami, M. Hajjaji

**Affiliations:** 1 occupational medecine, Hedi Chaker hospital, Sfax university; 2 occupational medecine; 3 Family department, University of Sfax; 4 rheumatology, Hedi Chaker hospital, Sfax university, Sfax, Tunisia

## Abstract

**Introduction:**

During the COVID-19 pandemic, there has been a peak of occupational dermatoses associated with Personal Protective Equipment (PPE) among healthcare workers (HCWs). The resulting dermatological damage might have an impact on the quality of life.

**Objectives:**

We aimed to evaluate the dermatological life quality of the HCWs due to PPE use.

**Methods:**

The study was conducted in a group of HCWs from Hedi Chaker hospital in Sfax, Tunisia. Data were gathered between march and may 2021 using a self-administered questionnaire including socio-professional characteristics, evaluation of skin lesions, evaluation of the infection-prevention practices and the Arabic version of the Dermatology Life Quality Index (DLQI).

**Results:**

Our sample was composed of 190 HCWs. The respondents’ mean age was 32.5 ± 6.5 years and 54.7% were female. The average job tenure was 6.6 ± 5.8 years. Doctors represented 22.1 %, nurses 13.6% and cleaning staff 64.3 % of participants. The prevalence of skin lesions due to PPE among HCWs was 51.6%. The mean time of mask use was 15.4 ± 9.1 hours. The daily hand washing frequency was >10 times/day in 85.3 % of participants. The mean DLQI score was 3.3 ± 4.3. According to this index, it was found that these skin lesions had no effect, small effects, moderate effects and very large effects on the lives of 43.9%, 34.7%, 12.2%, and 9.2% of participants, respectively. The DLQI was correlated with hand washing frequency (p = 0.014, r = 0.204) and the time of mask use ( p = 0.038, r = 0.172).

**Conclusions:**

Skin lesions among HCWs are frequent during the covid-19 pandemic. These lesions influence the quality of life of the HCWs. This risk gets higher with excessive preventive measures. It is critical to provide training on the prevention of skin lesions associated with PPE wearing and hand hygiene before and during the period of use of preventive measures.

**Disclosure of Interest:**

None Declared

